# Coronary Plaque Burden in South Asian Adults: Insights From the DILWALE Registry

**DOI:** 10.1016/j.jacadv.2026.103235

**Published:** 2026-09-18

**Authors:** Ahmed Mustafa Rashid, Sashwath Srikanth, Neeka Tavanaei, Swapnil Gupta, Kareena Patel, Tanushree Prasad, Jared Spitz, Priyanka Satish, Dinesh Kalra, Muhammad Shahzeb Khan, Amro Alsaid, Anandita Kulkarni

**Affiliations:** a Baylor Scott and White Research Institute, Baylor Scott and White Health, Dallas, Texas, USA; b Center for Cardiovascular Disease Prevention, Baylor Scott and White Health Heart Hospital Baylor-Plano, Plano, Texas, USA; c Inova Schar Heart and Vascular, Fairfax, Virginia, USA; d Center for Cardiovascular Prevention, Ascension Texas Cardiovascular, The University of Texas at Austin, Austin, Texas, USA; e Division of Cardiology, Department of Medicine, University of Louisville, Louisville, Kentucky, USA; f Department of Medicine, Baylor College of Medicine, Temple, Texas, USA

**Keywords:** CT angiogram, CVD, plaque, South Asian

## Abstract

**Background:**

South Asian (SA) adults have disproportionately high rates of premature coronary artery disease, yet conventional risk calculators may not fully capture coronary plaque burden.

**Objectives:**

We analyzed the DILWALE (DIL Wellness and Arterial Health Longitudinal Evaluation) registry to quantify coronary plaque burden using computed tomography angiography (CTA) and compare it across risk categories using the 10-year atherosclerotic cardiovascular disease (ASCVD) risk calculator and American Heart Association PREVENT-ASCVD Calculator.

**Methods:**

The DILWALE study is a medical record-based multicenter registry of SA adults (≥18 years) within Baylor Scott & White Health system. Patients who underwent clinically indicated coronary CTA were included and were risk stratified based on the PREVENT-ASCVD and Pooled Cohort Equations (PCE) risk scores. Quantitative plaque analysis was done using HeartFlow’s plaque analysis tool to evaluate the association among PCE, PREVENT, and plaque burden.

**Results:**

A total of 341 SAs were included. Among individuals stratified by the PREVENT score, median total plaque volume rose from 0 mm^3^ in the low-risk group (<5% 10-year predicted ASCVD risk) to 34 mm^3^, 161 mm^3^, and 161 mm^3^ in the borderline- (5%-7.4%), intermediate- (7.5%-19.9%), and high-risk (≥20%) groups, respectively (*P* < 0.0001). In the PCE cohort, median total plaque volume increased from 1 mm^3^ in the low-risk group to 91 mm^3^, 122 mm^3^, and 215 mm^3^ in the borderline-, intermediate-, and high-risk groups, respectively (*P* < 0.0001).

**Conclusions:**

In this SA cohort, higher PREVENT and ASCVD/PCE risk categories were associated with higher plaque burden. CTA reveals substantial noncalcified plaque missed by calcium scoring, underscoring its value for anatomic risk in SA adults.

South Asians (SAs) face a great burden of atherosclerotic cardiovascular disease (ASCVD), with higher rates of coronary disease and earlier onset—5 to 10 years sooner—than other populations.^1^ The risk of myocardial infarction (MI) and cardiovascular death in SA groups is nearly twice that observed in the European ancestry cohorts.^2^ This heightened vulnerability is multifactorial, involving genetic predisposition, high prevalence of metabolic risk factors, and environmental influences.^3^ The ASCVD Pooled Cohort Equations (PCE), which guide the 10-year risk estimation in U.S. prevention guidelines, were derived predominantly from White and Black American populations. Thus, traditional risk scores may not fully reflect the coronary plaque burden observed in SA adults.^1^ Recognizing these gaps, the American Heart Association (AHA) recently introduced the 2023 AHA “PREVENT” 10-year risk score (Predicting Risk of CVD Events).^4^ The PREVENT equations were explicitly designed to improve generalizability across diverse ethnic groups. In contrast to the PCE, the PREVENT model is sex-specific and omits race as a risk variable, acknowledging that race is a social construct rather than a biological determinant of risk.^5^^,^^6^

Coronary computed tomography angiography (CCTA) detects both calcified (CP) and noncalcified plaques (NCP), providing a comprehensive assessment of plaque burden.^7^ This is important because coronary artery calcium (CAC) scoring only identifies CP. Many SA patients—especially younger ones—may have noncalcified, lipid-rich plaque despite a CAC score of 0.^8^ Yet, they still face higher cardiac risk. Studies show that individuals with CAC = 0 may have NCP detectable on CCTA.^9^, ^10^, ^11^, ^12^ Thus, CCTA can identify subclinical atherosclerosis missed by calcium scoring alone. This is crucial for SAs, who often develop diffuse atherosclerosis.^8^

We used the DILWALE (DIL Wellness and Arterial Health Longitudinal Evaluation) registry—a multicenter electronic health record–derived cohort of SA American patients receiving care at Baylor Scott & White Health Center. The DILWALE registry provides a rich data set on demographics, risk factors, and clinical outcomes of SA adults in Texas.^3^^,^^13^ From this registry, we identified a subset of individuals who were clinically referred and underwent CCTA imaging. This referral-based subset may show higher plaque prevalence than asymptomatic or community-based SA cohorts. Using this clinically referred cohort, we sought to compare the 10-year ASCVD/PCE and PREVENT risk scores with CCTA plaque findings. Our aim was to evaluate the association between contemporary risk calculators and coronary plaque burden in SA patients, recognizing that risk prediction and disease detection represent related but distinct constructs.

## Methods

### Study design and population

We conducted a retrospective cohort study using the Baylor Scott & White DILWALE registry, which prospectively collects health data on SA patients across multiple centers.^13^ Eligible participants were SA adults (age ≥18 years) who underwent clinically indicated CCTA. SA ethnicity was self-identified from the Indian subcontinent (India, Pakistan, Bangladesh, Nepal, Sri Lanka, or neighboring countries). We included individuals without prior ASCVD, excluding those with any history of coronary artery disease (MI or revascularization), stroke, or peripheral arterial disease at the time of CCTA. This ensured a primary prevention cohort in which risk scores validated for patients without established ASCVD would apply. Patients with incomplete plaque data were excluded. The final study sample included SA patients with baseline CCTA and complete risk data to calculate 10-year ASCVD risk. This analysis included only clinically referred patients undergoing CCTA, which may limit generalizability to asymptomatic or community-based SA populations. Given the retrospective and noninterventional design of the study, the Baylor Scott & White Health Institutional Review Board granted a waiver of informed consent.

### Coronary CT angiography protocol

All CCTA studies used multidetector computed tomography (CT) scanners with standard cardiac imaging protocols. Scans were electrocardiogram-synchronized (prospective or retrospective gating) and obtained after intravenous iodinated contrast administration to visualize coronaries. Beta-blockers and sublingual nitroglycerin were used when appropriate to optimize image quality (target heart rate <65 beats/min and coronary vasodilation). Axial images were reconstructed at ∼0.5-mm slice thickness and were transferred to an advanced workstation for analysis. Coronary plaque quantification was carried out using an artificial intelligence (AI)–enabled software tool (HeartFlow Plaque Analysis, HeartFlow Inc). This Food and Drug Administration–cleared software generates a patient-specific 3-dimensional model of the coronary arterial lumen and wall from CCTA data. For each patient, we obtained total plaque (TP) volume (TPV), CP, and NCP volumes. The accuracy has been validated against intravascular ultrasound, showing high concordance for TPV and plaque subtypes.^14^ We classified individuals with TPV <20 mm^3^ and no visible stenosis as plaque-negative to minimize false-positive plaque detection from automated analysis. This approach aligns with prior studies that defined TPV <20 mm^3^ as a normal threshold and incorporated visual review of CAC = 0 cases to avoid misclassification of low-volume NCP.^15^ To further characterize plaque prevalence across alternative plaque definitions, we also reported plaque burden as TPV = 0 mm^3^ (no plaque), TPV >0 mm^3^ (any detectable plaque), TPV <20 mm^3^ (plaque below the prespecified threshold), and TPV ≥20 mm^3^ (plaque meeting the prespecified threshold).

### Risk factor and score assessment

Patient demographics and cardiovascular risk factors were extracted from the electronic medical record and registry data. Baseline variables included age, sex, blood pressure, cholesterol levels, diabetes history, smoking status, and medication use (eg, antihypertensives, statins). We used these variables to calculate two 10-year risk estimates for each individual: the American College of Cardiology (ACC)/AHA PCE (ASCVD Risk Estimator) and the AHA PREVENT 10-year CVD risk. PREVENT requires additional variables, including glycated hemoglobin and estimated glomerular filtration rate, that are not included in PCE. Consequently, fewer patients had complete data available for PREVENT calculation.

### Statistical analysis

We summarized the cohort’s characteristics using mean and SD for normally distributed continuous variables, median IQR for non-normally distributed continuous variables, and proportions for categorical variables. Plaque volumes, not normally distributed, were analyzed with nonparametric methods. The primary analysis compared coronary plaque burden across risk-score strata. Cohort was stratified by 10-year risk categories (low, borderline, intermediate, and high) for each risk estimator (ASCVD and PREVENT) and evaluated differences in plaque prevalence and extent. Chi-square test was used to compare prevalence of any plaque across risk categories. Kruskal-Wallis test compared plaque volumes between risk groups. As a sensitivity analysis, we compared coronary plaque prevalence between patients with and without calculable ASCVD/PCE and AHA PREVENT risk scores to assess the potential impact of missing risk-score components on plaque burden. Two-tailed tests with *P* < 0.05 were significant. Analyses used SAS 9.4 (SAS Institute).

## Results

### Baseline characteristics

A total of 341 SA patients who underwent clinically indicated CCTA were included (Table 1). Of these, 233 patients had sufficient data to calculate the 10-year AHA PREVENT risk score, 274 had sufficient data to calculate the 10-year ASCVD/PCE risk score, 209 had both risk scores available, and 43 had neither risk score available (Supplemental Table 1). Among patients with both risk scores available, agreement between ASCVD/PCE and AHA PREVENT risk categories was substantial (Kappa coefficient = 0.79; 95% CI:, 0.74-0.84).

**Table 1:** Baseline Characteristics of Patients Stratified by the AHA PREVENT and 10-Year ASCVD Risk Scores

	AHA PREVENT (n = 233)	10-Year ASCVD Risk (n = 274)
Age, y	54.0 (11.13)	55.0 (9.86)
Female	77 (33.0%)	86 (31.4%)
Smoking status		
Current	11 (4.7%)	12 (4.4%)
Former	26 (11.2%)	27 (9.9%)
Never	180 (77.3%)	214 (78.1%)
Unknown	15 (6.4%)	20 (7.3%)
BMI, kg/m^2^	26.8 (3.62)	27.1 (4.50)
Diabetes mellitus	63 (27.0%)	90 (32.8%)
Hyperlipidemia	195 (83.7%)	228 (83.2%)
Hypertension	132 (56.7%)	162 (59.1%)
LDL-C	111.7 (37.45)	105.5 (42.22)
HDL-C	48.9 (12.93)	48.1 (12.96)
Triglycerides^a^	125.0 (98.0, 169.5)	122.0 (92.0, 159.0)
Total cholesterol	188.1 (40.77)	179.6 (47.31)
Hemoglobin A_1c_	6.1 (1.16)	6.2 (1.19)
Statin therapy	126 (54.1%)	163 (59.5%)
eGFR	90.4 (16.70)	89.3 (16.10)

AHA = American Heart Association; ASCVD = atherosclerotic cardiovascular disease; BMI = body mass index; eGFR = estimated glomerular filtration rate; Hemoglobin A_1c_ = glycated hemoglobin; HDL-C = high-density lipoprotein cholesterol; LDL-C = low-density lipoprotein cholesterol.

a Median (IQR) reported.

The AHA PREVENT cohort comprising 233 eligible patients had a mean age of 54 years and 33% (n = 77) were women. Most participants were never smokers (77%, n = 180), followed by former (11%, n = 26) and current smokers (4.7%, n = 11). Hyperlipidemia was the most common comorbidity (84%, n = 195), followed by hypertension (57%, n = 132) and diabetes (27%, n = 63). Statin therapy was prescribed in 54% (n = 126) of individuals. Based on the AHA PREVENT 10-year risk, 50% (n = 116) were at low risk, 14% (n = 32) were at borderline risk, 30% (n = 69) were at intermediate risk, and 7% (n = 16) were at high risk. Based on the PREVENT 30-year risk, 1% (n = 2) were at low risk, 3% (n = 6) were at borderline risk, 31% (n = 72) were at intermediate risk, and 66% (n = 153) were at high risk.

The 10-year ASCVD cohort comprising 274 eligible patients had a mean age of 55 years and 31% (n = 86) were women. Most participants were never smokers (78%, n = 214), followed by former (10%, n = 27) and current smokers (4.4%, n = 12). Hyperlipidemia was the most common comorbidity (83%, n = 228), followed by hypertension (59%, n = 162) and diabetes (33%, n = 90). Statin therapy was prescribed in 60% (n = 163) of individuals. Based on the ASCVD 10-year risk, 47% (n = 128) were at low risk, 15% (n = 40) were at borderline risk, 24% (n = 65) were at intermediate risk, and 15% (n = 41) were at high risk.

Statin use was 52%, 55%, 66%, and 76% across low-, borderline-, intermediate-, and high-risk ASCVD groups, respectively. Statin use was 44%, 50%, 68%, and 75% across low-, borderline-, intermediate-, and high-risk individuals as characterized by the 10-year AHA PREVENT risk score, respectively. Statin-stratified plaque prevalence generally increased across risk categories under all four alternative TPV definitions, with similar patterns observed among CAC = 0 subgroups (Table 2).

**Table 2:** Plaque Prevalence Stratified by Statin Use Across 10-Year ASCVD/PCE and 10- and 30-Year AHA PREVENT Risk Categories in the Overall Cohort and Among Patients With a Calcium Score of 0 AU

Statin Use	Plaque Burden	Total	Low Risk (<5%)	Borderline Risk (5%–7.4%)	Intermediate Risk (7.5%–19.9%)	High Risk (≥20%)	*P* Value
10-y ASCVD/PCE risk: overall		(n = 111)	(n = 61)	(n = 18)	(n = 22)	(n = 10)	
No statin	TPV = 0 mm^3^	48 (43.2%)	41 (67.2%)	4 (22.2%)	3 (13.6%)	0 (0.0%)	**<0.0001**
No statin	TPV >0 mm^3^	63 (56.8%)	20 (32.8%)	14 (77.8%)	19 (86.4%)	10 (100.0%)	
No statin	TPV <20 mm^3^	60 (54.1%)	49 (80.3%)	4 (22.2%)	6 (27.3%)	1 (10.0%)	**<0.0001**
No statin	TPV ≥20 mm^3^	51 (45.9%)	12 (19.7%)	14 (77.8%)	16 (72.7%)	9 (90.0%)	
10-y ASCVD/PCE risk: overall		(n = 163)	(n = 67)	(n = 22)	(n = 43)	(n = 31)	
Statin use	TPV = 0 mm^3^	33 (20.2%)	22 (32.8%)	5 (22.7%)	5 (11.6%)	1 (3.2%)	**0.0026**
Statin use	TPV >0 mm^3^	130 (79.8%)	45 (67.2%)	17 (77.3%)	38 (88.4%)	30 (96.8%)	
Statin use	TPV <20 mm^3^	45 (27.6%)	27 (40.3%)	9 (40.9%)	6 (14.0%)	3 (9.7%)	**0.0010**
Statin use	TPV ≥20 mm^3^	118 (72.4%)	40 (59.7%)	13 (59.1%)	37 (86.0%)	28 (90.3%)	
10-y ASCVD/PCE risk: CAC = 0		(n = 60)	(n = 50)	(n = 4)	(n = 5)	(n = 1)	
No statin	TPV = 0 mm^3^	43 (71.7%)	38 (76.0%)	3 (75.0%)	2 (40.0%)	0 (0.0%)	0.0921
No statin	TPV >0 mm^3^	17 (28.3%)	12 (24.0%)	1 (25.0%)	3 (60.0%)	1 (100.0%)	
No statin	TPV <20 mm^3^	54 (90.0%)	45 (90.0%)	3 (75.0%)	5 (100.0%)	1 (100.0%)	0.4759
No statin	TPV ≥20 mm^3^	6 (10.0%)	5 (10.0%)	1 (25.0%)	0 (0.0%)	0 (0.0%)	
10-y ASCVD/PCE risk: CAC = 0		(n = 40)	(n = 26)	(n = 8)	(n = 6)	(n = 0)	
Statin use	TPV = 0 mm^3^	28 (70.0%)	19 (73.1%)	5 (62.5%)	4 (66.7%)	0 (0.0%)	0.8339
Statin use	TPV >0 mm^3^	12 (30.0%)	7 (26.9%)	3 (37.5%)	2 (33.3%)	0 (0.0%)	
Statin use	TPV <20 mm^3^	35 (87.5%)	23 (88.5%)	8 (100.0%)	4 (66.7%)	0 (0.0%)	0.1698
Statin use	TPV ≥20 mm^3^	5 (12.5%)	3 (11.5%)	0 (0.0%)	2 (33.3%)	0 (0.0%)	
10-y AHA PREVENT risk: overall		(n = 107)	(n = 65)	(n = 16)	(n = 22)	(n = 4)	
No statin	TPV = 0 mm^3^	48 (44.9%)	42 (64.6%)	4 (25.0%)	2 (9.1%)	0 (0.0%)	**<0.0001**
No statin	TPV >0 mm^3^	59 (55.1%)	23 (35.4%)	12 (75.0%)	20 (90.9%)	4 (100.0%)	
No statin	TPV <20 mm^3^	63 (58.9%)	51 (78.5%)	6 (37.5%)	4 (18.2%)	2 (50.0%)	**<0.0001**
No statin	TPV ≥20 mm^3^	44 (41.1%)	14 (21.5%)	10 (62.5%)	18 (81.8%)	2 (50.0%)	
10-y AHA PREVENT risk: overall		(n = 126)	(n = 51)	(n = 16)	(n = 47)	(n = 12)	
Statin use	TPV = 0 mm^3^	32 (25.4%)	21 (41.2%)	5 (31.3%)	6 (12.8%)	0 (0.0%)	**0.0006**
Statin use	TPV >0 mm^3^	94 (74.6%)	30 (58.8%)	11 (68.8%)	41 (87.2%)	12 (100.0%)	
Statin use	TPV <20 mm^3^	39 (31.0%)	23 (45.1%)	8 (50.0%)	8 (17.0%)	0 (0.0%)	**0.0003**
Statin use	TPV ≥20 mm^3^	87 (69.0%)	28 (54.9%)	8 (50.0%)	39 (83.0%)	12 (100.0%)	
10-y AHA PREVENT risk: CAC = 0		(n = 62)	(n = 52)	(n = 5)	(n = 3)	(n = 2)	
No statin	TPV = 0 mm^3^	43 (69.4%)	39 (75.0%)	3 (60.0%)	1 (33.3%)	0 (0.0%)	0.0524
No statin	TPV >0 mm^3^	19 (30.6%)	13 (25.0%)	2 (40.0%)	2 (66.7%)	2 (100.0%)	
No statin	TPV <20 mm^3^	56 (90.3%)	46 (88.5%)	5 (100.0%)	3 (100.0%)	2 (100.0%)	1.0000
No statin	TPV ≥20 mm^3^	6 (9.7%)	6 (11.5%)	0 (0.0%)	0 (0.0%)	0 (0.0%)	
10-y AHA PREVENT risk: CAC = 0		(n = 34)	(n = 21)	(n = 8)	(n = 5)	(n = 0)	
Statin use	TPV = 0 mm^3^	27 (79.4%)	18 (85.7%)	5 (62.5%)	4 (80.0%)	0 (0.0%)	0.3847
Statin use	TPV >0 mm^3^	7 (20.6%)	3 (14.3%)	3 (37.5%)	1 (20.0%)	0 (0.0%)	
Statin use	TPV <20 mm^3^	31 (91.2%)	19 (90.5%)	8 (100.0%)	4 (80.0%)	0 (0.0%)	0.4576
Statin use	TPV ≥20 mm^3^	3 (8.8%)	2 (9.5%)	0 (0.0%)	1 (20.0%)	0 (0.0%)	
30-y AHA PREVENT risk: overall		(n = 107)	(n = 2)	(n = 3)	(n = 48)	(n = 54)	
No statin	TPV = 0 mm^3^	48 (44.9%)	0 (0.0%)	2 (66.7%)	36 (75.0%)	10 (18.5%)	**<0.0001**
No statin	TPV >0 mm^3^	59 (55.1%)	2 (100.0%)	1 (33.3%)	12 (25.0%)	44 (81.5%)	
No statin	TPV <20 mm^3^	63 (58.9%)	1 (50.0%)	2 (66.7%)	43 (89.6%)	17 (31.5%)	**<0.0001**
No statin	TPV ≥20 mm^3^	44 (41.1%)	1 (50.0%)	1 (33.3%)	5 (10.4%)	37 (68.5%)	
30-y AHA PREVENT risk: overall		(n = 126)	(n = 0)	(n = 3)	(n = 24)	(n = 99)	
Statin use	TPV = 0 mm^3^	32 (25.4%)	0 (0.0%)	3 (100.0%)	8 (33.3%)	21 (21.2%)	**0.0052**
Statin use	TPV >0 mm^3^	94 (74.6%)	0 (0.0%)	0 (0.0%)	16 (66.7%)	78 (78.8%)	
Statin use	TPV <20 mm^3^	39 (31.0%)	0 (0.0%)	3 (100.0%)	9 (37.5%)	27 (27.3%)	**0.0202**
Statin use	TPV ≥20 mm^3^	87 (69.0%)	0 (0.0%)	0 (0.0%)	15 (62.5%)	72 (72.7%)	
30-y AHA PREVENT risk: CAC = 0		(n = 62)	(n = 2)	(n = 2)	(n = 40)	(n = 18)	
No statin	TPV = 0 mm^3^	43 (69.4%)	0 (0.0%)	2 (100.0%)	33 (82.5%)	8 (44.4%)	**0.0013**
No statin	TPV >0 mm^3^	19 (30.6%)	2 (100.0%)	0 (0.0%)	7 (17.5%)	10 (55.6%)	
No statin	TPV <20 mm^3^	56 (90.3%)	1 (50.0%)	2 (100.0%)	38 (95.0%)	15 (83.3%)	0.0983
No statin	TPV ≥20 mm^3^	6 (9.7%)	1 (50.0%)	0 (0.0%)	2 (5.0%)	3 (16.7%)	
30-y AHA PREVENT risk: CAC = 0		(n = 34)	(n = 0)	(n = 3)	(n = 8)	(n = 23)	
Statin use	TPV = 0 mm^3^	27 (79.4%)	0 (0.0%)	3 (100.0%)	8 (100.0%)	16 (69.6%)	0.1215
Statin use	TPV >0 mm^3^	7 (20.6%)	0 (0.0%)	0 (0.0%)	0 (0.0%)	7 (30.4%)	
Statin use	TPV <20 mm^3^	31 (91.2%)	0 (0.0%)	3 (100.0%)	8 (100.0%)	20 (87.0%)	0.4553
Statin use	TPV ≥20 mm^3^	3 (8.8%)	0 (0.0%)	0 (0.0%)	0 (0.0%)	3 (13.0%)	

Values are n (%).

TPV = 0 mm^3^ indicates that no plaque is present; TPV >0 mm^3^ indicates the presence of plaque; TPV <20 mm^3^ indicates detectable plaque below the predefined study threshold; TPV ≥20 mm^3^ indicates plaque meeting the predefined threshold used in the primary analysis.

**Bold** value indicates statistically significant *P* values (*P* < 0.05).

CAC = coronary artery calcium; PCE = Pooled Cohort Equations; TPV = total plaque volume; other abbreviations as in Table 1.

### CAC scores

CAC score distribution varied significantly across PREVENT and ASCVD 10-year risk categories (each *P* < 0.0001) (Figures 1 and 2, respectively). Overall, the proportion with CAC = 0 decreased progressively with higher risk categories, whereas CAC ≥100 increased. In the PREVENT cohort, 66% of low-risk individuals had CAC = 0 compared with 13% in the high-risk group, whereas CAC ≥100 was present in only 11% of low-risk vs 63% of high-risk participants. Similar gradients were observed for ASCVD risk, where 62% of low-risk and 3% of high-risk individuals had CAC = 0, and CAC ≥100 increased from 14% in low-risk to 62% in high-risk group.

**Figure 1:**
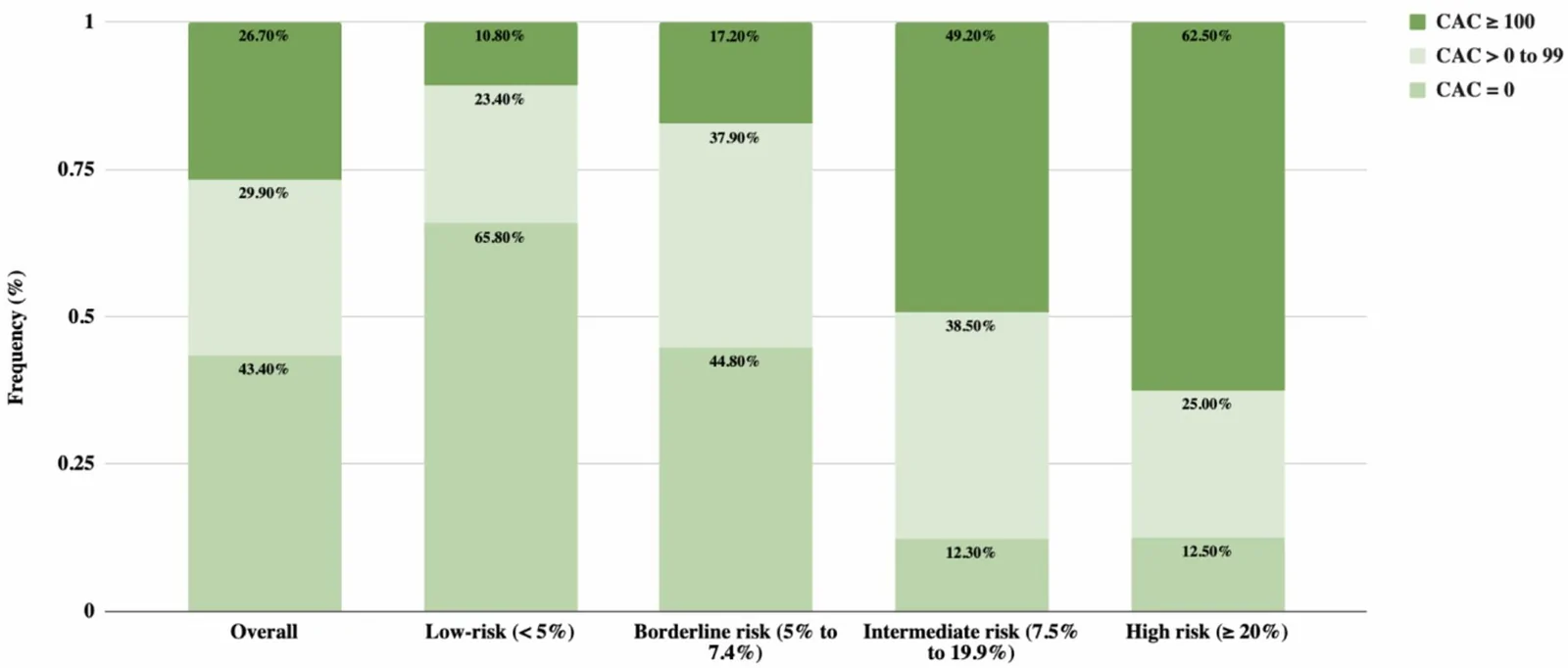
Distribution of Coronary Artery Calcium Among Patients Stratified by the 10-Year AHA PREVENT Calculator Figure showing a graded decline in the proportion with CAC = 0 and a reciprocal rise in CAC ≥100 with increasing PREVENT-risk. CAC = coronary artery calcium.

**Figure 2:**
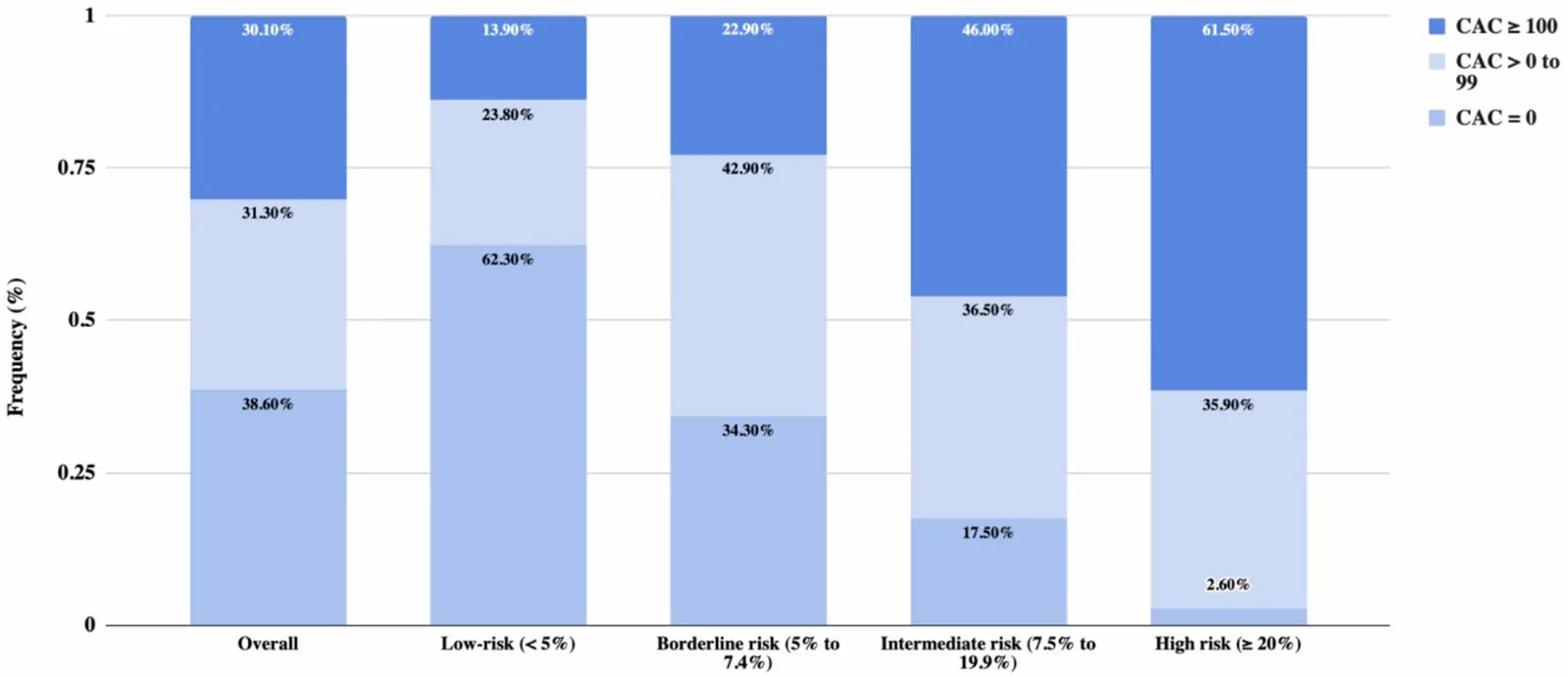
Distribution of Coronary Artery Calcium Score Among Patients Stratified by the 10-Year Atherosclerotic Cardiovascular Disease/Pooled Cohort Equations Risk Score Figure showing a graded decline in the proportion with CAC = 0 and a reciprocal rise in CAC ≥100 with increasing atherosclerotic cardiovascular disease/Pooled Cohort Equations risk. Abbreviation as in Figure 1.

### Plaque burden across AHA PREVENT risk categories

Among SA adults, the presence of coronary plaque increased stepwise with higher PREVENT 10-year risk group, from 36% in the low-risk to 56% in borderline-risk, 83% in intermediate-risk, and 88% in high-risk individuals (Table 3) (*P* < 0.0001). The median TPV was greater across higher PREVENT risk groups, from 0.0 mm^3^ (0.0, 44.5) in low-risk to 34.0 mm^3^ (0.0, 221.0) in borderline-risk, 161.0 mm^3^ (47.0, 530.0) in intermediate-risk, and 161.0 mm^3^ (47.0, 530.0) in high-risk patients (*P* < 0.0001). Similar overall trends were observed in CP, low-attenuation plaque (LAP), and NCP volumes. The median CP volumes increased from 0.0 mm^3^ (0.0, 3.0) in low-risk to 0.5 mm^3^ (0.0, 28.0) in borderline-risk, 22.0 mm^3^ (6.0, 114.0) in intermediate-risk, and 20.0 mm^3^ (2.0, 112.0) in high-risk patients (*P* < 0.0001). NCP increased from 0.0 mm^3^ (0.0, 37.5) in low-risk to 29.0 (0.0, 196.0) in borderline-risk, 138.0 (41.0, 416.0) in intermediate-risk, and 130.0 mm^3^ (89.5, 488.0) in high-risk groups (*P* < 0.0001). LAP increased from 0.0 mm^3^ (0.0, 1.0) in low-risk to 1.0 mm^3^ (0.0, 3.0) in borderline-risk, 2.0 mm^3^ (0.0, 5.0) in intermediate-risk, and 1.0 mm^3^ (0.0, 4.5) in high-risk groups (*P* < 0.0001). The burden of high-risk LAP (≥10 mm^3^) increased from 6% in low-risk to 21% in borderline-risk and 34% in intermediate-risk and was 0% in high-risk patients (*P* = 0.0007). Analyses using the 30-year PREVENT score demonstrated comparable trends, with the highest burden of CP, NCP, and LAP observed in high-risk categories (Supplemental Table 2).

**Table 3:** Presence of Plaque and Plaque Subtypes Among Patients Stratified by the 10-Y AHA PREVENT Calculator

	10-Year AHA PREVENT Risk				*P* Value
	Low Risk (<5%) (n = 116)	Borderline Risk (5%–7.4%) (n = 32)	Intermediate Risk (7.5%–19.9%) (n = 69)	High Risk (≥ 20%) (n = 16)	
Overall coronary arteries					
Presence of plaque, n (%)	42 (36%)	18 (56%)	57 (83%)	14 (88%)	**<0.0001**
Total plaque = 0 mm^3^, n (%)	63 (54.3%)	9 (28.1%)	8 (11.6%)	0 (0.0%)	**<0.0001**
Total plaque >0 mm^3^, n (%)	53 (45.7%)	23 (71.9%)	61 (88.4%)	16 (100.0%)	**<0.0001**
Total plaque <20 mm^3^, n (%)	74 (63.8%)	14 (43.8%)	12 (17.4%)	2 (12.5%)	**<0.0001**
Total plaque ≥20 mm^3^, n (%)	42 (36.2%)	18 (56.2%)	57 (82.6%)	14 (87.5%)	**<0.0001**
Total plaque	0.0 (0.0, 44.5)	34.0 (0.0, 221.0)	161.0 (47.0, 530.0)	161.0 (47.0, 530.0)	**<0.0001**
Calcified plaque	0.0 (0.0, 3.0)	0.5 (0.0, 28.0)	22.0 (6.0, 114.0)	20.0 (2.0, 112.0)	**<0.0001**
Low-attenuation plaque	0.0 (0.0, 1.0)	1.0 (0.0, 3.0)	2.0 (0.0, 5.0)	1.0 (0.0, 4.5)	**<0.0001**
High-risk low-attenuation plaque (≥10 mm^3^), n (%)	5 (5.7%)	4 (21.1%)	12 (34.3%)	0 (0.0%)	**0.0007**
Noncalcified plaque	0.0 (0.0, 37.5)	29.0 (0.0, 196.0)	138.0 (41.0, 416.0)	130.0 (89.5, 488.0)	**<0.0001**
Left main					
Total plaque	0.0 (0.0, 0.0)	0.0 (0.0, 8.5)	0.0 (0.0, 40.0)	40.5 (0.0, 57.5)	**<0.0001**
Calcified plaque	0.0 (0.0, 0.0)	0.0 (0.0, 0.0)	0.0 (0.0, 3.0)	7.0 (0.0, 16.5)	**<0.0001**
Low-attenuation plaque	0.0 (0.0, 0.0)	0.0 (0.0, 0.0)	0.0 (0.0, 0.0)	0.0 (0.0, 0.5)	**0.0021**
Noncalcified plaque	0.0 (0.0, 0.0)	0.0 (0.0, 7.5)	0.0 (0.0, 35.0)	34.0 (0.0, 41.0)	**<0.0001**
Left anterior descending artery					
Total plaque	0.0 (0.0, 15.5)	29.5 (0.0, 114.5)	76.0 (11.0, 209.0)	63.5 (0.0, 272.5)	**<0.0001**
Calcified plaque	0.0 (0.0, 0.0)	0.0 (0.0, 16.5)	10.0 (1.0, 33.0)	4.5 (0.0, 88.5)	**<0.0001**
Low-attenuation plaque	0.0 (0.0, 0.0)	0.5 (0.0, 3.0)	1.0 (0.0, 3.0)	1.0 (0.0, 2.0)	**<0.0001**
Noncalcified plaque	0.0 (0.0, 14.0)	26.0 (0.0, 100.5)	57.0 (11.0, 175.0)	58.0 (0.0, 185.5)	**<0.0001**
Left circumflex artery					-
Total plaque	0.0 (0.0, 0.5)	0.0 (0.0, 26.0)	35.0 (0.0, 85.0)	47.0 (16.5, 117.0)	
Calcified plaque	0.0 (0.0, 0.0)	0.0 (0.0, 0.5)	2.0 (0.0, 13.0)	5.0 (0.0, 22.0)	**<0.0001**
Low-attenuation plaque	0.0 (0.0, 0.0)	0.0 (0.0, 0.0)	0.0 (0.0, 1.0)	0.0 (0.0, 1.0)	**0.0012**
Noncalcified plaque	0.0 (0.0, 0.5)	0.0 (0.0, 25.0)	28.0 (0.0, 75.0)	43.0 (14.0, 92.5)	**<0.0001**
Right coronary artery					
Total plaque	0.0 (0.0, 0.0)	0.0 (0.0, 20.5)	24.0 (0.0, 143.0)	25.0 (12.0, 145.0)	**<0.0001**
Calcified plaque	0.0 (0.0, 0.0)	0.0 (0.0, 3.0)	1.0 (0.0, 13.0)	2.0 (0.0, 18.0)	**<0.0001**
Low-attenuation plaque	0.0 (0.0, 0.0)	0.0 (0.0, 0.0)	0.0 (0.0, 1.0)	0.0 (0.0, 1.0)	**0.0118**
Noncalcified plaque	0.0 (0.0, 0.0)	0.0 (0.0, 19.0)	21.0 (0.0, 123.0)	22.5 (9.5, 128.0)	**<0.0001**

Median (IQR), plaque volume is reported in mm^3^.

**Bold** value indicates statistically significant *P* values (*P* < 0.05).

Abbreviation as in Table 1.

### Plaque burden across 10-year ASCVD/PCE risk categories

Among SA adults, the presence of coronary plaque increased stepwise with higher ASCVD 10-year risk group, from 41% in the low-risk group to 68% in borderline-risk, 82% in intermediate-risk, and 90% in high-risk individuals (Table 4) (*P* < 0.0001). The median TPV increased progressively across 10-year ASCVD risk categories, from 1.0 mm^3^ (0.0, 55.5) in low-risk to 91.0 mm^3^ (3.5, 439.0) in borderline-risk, 122.0 mm^3^ (24.0, 470.0) in intermediate-risk, and 215.0 mm^3^ (88.0, 716.0) in high-risk patients (*P* < 0.0001). Similar trends were observed in CP, LAP, and NCP volumes. The median CP volumes increased from 0.0 mm^3^ (0.0, 9.0) in low-risk to 6.5 mm^3^ (0.0, 81.5) in borderline-risk, 18.0 mm^3^ (1.0, 79.0) in intermediate-risk, and 30.0 mm^3^ (16.0, 165.0) in high-risk patients (*P* < 0.0001). The median NCP increased from 1.0 (0.0, 50.5) mm^3^ in low-risk to 79.0 mm^3^ (3.5, 357.5) in borderline-risk, 91.0 mm^3^ (22.0, 361.0) in intermediate-risk, and 185.0 mm^3^ (73.0, 518.0) in high-risk groups (*P* < 0.0001). LAP increased from 0.0 mm^3^ (0.0, 1.0) in low-risk to 1.0 mm^3^ (0.0, 3.0) in borderline-risk, 2.0 mm^3^ (0.0, 4.0) in intermediate-risk, and 2.0 mm^3^ (0.0, 6.0) in high-risk groups (*P* < 0.0001). The burden of high-risk LAP (≥10 mm^3^) increased from 7.7% in low-risk to 27.3% in borderline-risk and 25% in intermediate-risk and was 45% in high-risk patients (*P* < 0.0001).

**Table 4:** Presence of Plaque and Plaque Subtypes Among Patients Stratified by the 10-Year ASCVD/PCE Risk Score

	10-Year ASCVD/PCE Risk				*P* Value
	Low Risk (<5%) (n = 128)	Borderline Risk (5%–7.4%) (n = 40)	Intermediate Risk (7.5%–19.9%) (n = 65)	High Risk (≥ 20%) (n = 41)	
Overall coronary arteries					
Presence of plaque, n (%)	52 (41%)	27 (68%)	53 (82%)	37 (90%)	**<0.0001**
Total plaque = 0 mm^3^, n (%)	63 (49.2%)	9 (22.5%)	8 (12.3%)	1 (2.4%)	**<0.0001**
Total plaque >0 mm^3^, n (%)	65 (50.8%)	31 (77.5%)	57 (87.7%)	40 (97.6%)	**<0.0001**
Total plaque <20 mm^3^, n (%)	76 (59.4%)	13 (32.5%)	12 (18.5%)	4 (9.8%)	**<0.0001**
Total plaque ≥20 mm^3^, n (%)	52 (40.6%)	27 (67.5%)	53 (81.5%)	37 (90.2%)	**<0.0001**
Total plaque	1.0 (0.0, 55.5)	91.0 (3.5, 439.0)	122.0 (24.0, 470.0)	215.0 (88.0, 716.0)	**<0.0001**
Calcified plaque	0.0 (0.0, 9.0)	6.5 (0.0, 81.5)	18.0 (1.0, 79.0)	30.0 (16.0, 165.0)	**<0.0001**
Low-attenuation plaque	0.0 (0.0, 1.0)	1.0 (0.0, 3.0)	2.0 (0.0, 4.0)	2.0 (0.0, 6.0)	**<0.0001**
High-risk, low-attenuation plaque (≥10 mm^3^)	7 (7.7%)	6 (27.3%)	8 (25.0%)	9 (45.0%)	**<0.0001**
Noncalcified plaque	1.0 (0.0, 50.5)	79.0 (3.5, 357.5)	91.0 (22.0, 361.0)	185.0 (73.0, 518.0)	**<0.0001**
Left main					
Total plaque	0.0 (0.0, 0.0)	0.0 (0.0, 10.5)	0.0 (0.0, 20.0)	26.0 (0.0, 58.0)	**<0.0001**
Calcified plaque	0.0 (0.0, 0.0)	0.0 (0.0, 0.0)	0.0 (0.0, 3.0)	3.0 (0.0, 14.0)	**<0.0001**
Low-attenuation plaque	0.0 (0.0, 0.0)	0.0 (0.0, 0.0)	0.0 (0.0, 0.0)	0.0 (0.0, 1.0)	**<0.0001**
Noncalcified plaque	0.0 (0.0, 0.0)	0.0 (0.0, 10.0)	0.0 (0.0, 13.0)	20.0 (0.0, 47.0)	**<0.0001**
Left anterior descending artery					
Total plaque	0.0 (0.0, 31.0)	43.0 (0.0, 235.0)	72.0 (11.0, 163.0)	61.0 (8.0, 226.0)	**<0.0001**
Calcified plaque	0.0 (0.0, 2.0)	4.0 (0.0, 55.5)	6.0 (0.0, 32.0)	11.0 (2.0, 38.0)	**<0.0001**
Low-attenuation plaque	0.0 (0.0, 0.5)	1.0 (0.0, 2.0)	1.0 (0.0, 3.0)	1.0 (0.0, 3.0)	**<0.0001**
Noncalcified plaque	0.0 (0.0, 28.5)	37.0 (0.0, 172.5)	61.0 (11.0, 130.0)	53.0 (8.0, 194.0)	**<0.0001**
Left circumflex artery					
Total plaque	0.0 (0.0, 4.0)	2.0 (0.0, 61.5)	3.0 (0.0, 95.0)	49.0 (5.0, 79.0)	**<0.0001**
Calcified plaque	0.0 (0.0, 0.0)	0.0 (0.0, 8.0)	0.0 (0.0, 13.0)	5.0 (0.0, 13.0)	**<0.0001**
Low-attenuation plaque	0.0 (0.0, 0.0)	0.0 (0.0, 0.0)	0.0 (0.0, 1.0)	0.0 (0.0, 1.0)	**0.0008**
Noncalcified plaque	0.0 (0.0, 4.0)	2.0 (0.0, 51.5)	3.0 (0.0, 79.0)	46.0 (3.0, 73.0)	**<0.0001**
Right coronary artery					
Total plaque	0.0 (0.0, 3.0)	2.5 (0.0, 48.5)	16.0 (0.0, 103.0)	21.0 (0.0, 228.0)	**<0.0001**
Calcified plaque	0.0 (0.0, 0.0)	0.0 (0.0, 3.0)	0.0 (0.0, 10.0)	1.0 (0.0, 45.0)	**<0.0001**
Low-attenuation plaque	0.0 (0.0, 0.0)	0.0 (0.0, 0.5)	0.0 (0.0, 1.0)	0.0 (0.0, 2.0)	**0.0054**
Noncalcified plaque	0.0 (0.0, 3.0)	2.5 (0.0, 46.0)	16.0 (0.0, 93.0)	19.0 (0.0, 203.0)	**<0.0001**

Median (IQR), plaque volume is reported in mm^3^

**Bold** value indicates statistically significant *P* values (*P* < 0.05).

Abbreviations as in Tables 1 and 2.

### Plaque burden by risk score availability

In the complete HeartFlow cohort, plaque prevalence did not differ significantly by ASCVD data availability for either any detectable plaque (TPV >0 mm^3^; 77.6% vs 70.4%; *P* = 0.2418) or TPV ≥20 mm^3^ (68.7% vs 61.7%; *P* = 0.2888). In contrast, patients missing PREVENT data had higher plaque prevalence than those with PREVENT data for both TPV >0 mm^3^ (85.2% vs 65.7%; *P* = 0.0002) and TPV ≥20 mm^3^ (77.8% vs 56.2%; *P* = 0.0001). These findings were similar in the sensitivity analysis that excluded the 43 patients missing both scores, with persistent significance for 10-year AHA PREVENT but not for 10-year ASCVD risk group (Supplemental Table 3).

### Plaque burden among individuals with CAC = 0

#### 10-year PREVENT and ASCVD cohorts

A total of 96 and 100 patients in the 10-year PREVENT and 10-year ASCVD cohorts had CAC = 0, respectively (Table 5 and 6). Plaque volumes were low overall across low-, borderline-, and intermediate-risk groups.

**Table 5:** Presence of Plaque and Plaque Subtypes Among Patients With a Calcium Score of 0 AU Stratified by the 10-Year AHA PREVENT Calculator

	10-Year AHA PREVENT Risk				*P* Value
	Low Risk (<5%) (n = 73)	Borderline Risk (5%–7.4%) (n = 13)	Intermediate Risk (7.5%–19.9%) (n = 8)	High Risk (≥ 20%) (n = 2)	
Overall coronary arteries					
Presence of plaque, n (%)	8 (11.0%)	0 (0.0%)	1 (12.5%)	0 (0.0%)	0.6316
Total plaque = 0 mm^3^, n (%)	57 (78.1%)	8 (61.5%)	5 (62.5%)	0 (0.0%)	0.058
Total plaque >0 mm^3^, n (%)	16 (21.9%)	5 (38.5%)	3 (37.5%)	2 (100.0%)	0.058
Total plaque <20 mm^3^, n (%)	65 (89.0%)	13 (100.0%)	7 (87.5%)	2 (100.0%)	0.6316
Total plaque ≥20 mm^3^, n (%)	8 (11.0%)	0 (0.0%)	1 (12.5%)	0 (0.0%)	0.6316
Total plaque	0.0 (0.0, 0.0)	0.0 (0.0, 3.0)	0.0 (0.0, 1.5)	7.5 (4.0, 11.0)	0.134
Calcified plaque	0.0 (0.0, 0.0)	0.0 (0.0, 0.0)	0.0 (0.0, 0.0)	0.0 (0.0, 0.0)	0.888
Low-attenuation plaque	0.0 (0.0, 0.0)	0.0 (0.0, 0.0)	0.0 (0.0, 0.5)	0.0 (0.0, 0.0)	0.5865
High-risk low-attenuation plaque (≥10 mm^3^)	1 (1.5%)	0 (0.0%)	0 (0.0%)	0 (0.0%)	1.0000
Noncalcified plaque	0.0 (0.0, 0.0)	0.0 (0.0, 3.0)	0.0 (0.0, 1.5)	7.5 (4.0, 11.0)	0.134
Left main					
Total plaque	0.0 (0.0, 0.0)	0.0 (0.0, 0.0)	0.0 (0.0, 0.0)	0.0 (0.0, 0.0)	0.9572
Calcified plaque	0.0 (0.0, 0.0)	0.0 (0.0, 0.0)	0.0 (0.0, 0.0)	0.0 (0.0, 0.0)	1.0000
Low-attenuation plaque	0.0 (0.0, 0.0)	0.0 (0.0, 0.0)	0.0 (0.0, 0.0)	0.0 (0.0, 0.0)	1.0000
Noncalcified plaque	0.0 (0.0, 0.0)	0.0 (0.0, 0.0)	0.0 (0.0, 0.0)	0.0 (0.0, 0.0)	0.9572
Left anterior descending artery					
Total plaque	0.0 (0.0, 0.0)	0.0 (0.0, 0.0)	0.0 (0.0, 1.0)	0.0 (0.0, 0.0)	0.6848
Calcified plaque	0.0 (0.0, 0.0)	0.0 (0.0, 0.0)	0.0 (0.0, 0.0)	0.0 (0.0, 0.0)	1.0000
Low-attenuation plaque	0.0 (0.0, 0.0)	0.0 (0.0, 0.0)	0.0 (0.0, 0.5)	0.0 (0.0, 0.0)	0.0741
Noncalcified plaque	0.0 (0.0, 0.0)	0.0 (0.0, 0.0)	0.0 (0.0, 1.0)	0.0 (0.0, 0.0)	0.6848
Left circumflex artery					
Total plaque	0.0 (0.0, 0.0)	0.0 (0.0, 0.0)	0.0 (0.0, 0.0)	0.0 (0.0, 0.0)	0.6497
Calcified plaque	0.0 (0.0, 0.0)	0.0 (0.0, 0.0)	0.0 (0.0, 0.0)	0.0 (0.0, 0.0)	1.0000
Low-attenuation plaque	0.0 (0.0, 0.0)	0.0 (0.0, 0.0)	0.0 (0.0, 0.0)	0.0 (0.0, 0.0)	0.9572
Noncalcified plaque	0.0 (0.0, 0.0)	0.0 (0.0, 0.0)	0.0 (0.0, 0.0)	0.0 (0.0, 0.0)	0.6497
Right coronary artery					
Total plaque	0.0 (0.0, 0.0)	0.0 (0.0, 0.0)	0.0 (0.0, 0.0)	7.5 (4.0, 11.0)	**0.0010**
Calcified plaque	0.0 (0.0, 0.0)	0.0 (0.0, 0.0)	0.0 (0.0, 0.0)	0.0 (0.0, 0.0)	0.8880
Low-attenuation plaque	0.0 (0.0, 0.0)	0.0 (0.0, 0.0)	0.0 (0.0, 0.0)	0.0 (0.0, 0.0)	0.6817
Noncalcified plaque	0.0 (0.0, 0.0)	0.0 (0.0, 0.0)	0.0 (0.0, 0.0)	7.5 (4.0, 11.0)	**0.0009**

Median (IQR), plaque volume is reported in mm^3^.

**Bold** value indicates statistically significant *P* values (*P* < 0.05).

Abbreviation as in Table 1.

**Table 6:** Presence of Plaque and Plaque Subtypes Among Patients With a Calcium Score of 0 Agatston Units Stratified by the 10-Year ASCVD/PCE Risk Score

	10-Year ASCVD/PCE Risk Score				*P* Value
	Low Risk (<5%) (n = 76)	Borderline Risk (5%–7.4%) (n = 12)	Intermediate Risk (7.5%–19.9%) (n = 11)	High Risk (≥ 20%) (n = 1)	
Overall coronary arteries					
Presence of plaque, n (%)	8 (10.5%)	1 (8.3%)	2 (18.2%)	0 (0.0%)	0.7699
Total plaque = 0 mm^3^, n (%)	57 (75.0%)	8 (66.7%)	6 (54.6%)	0 (0.0%)	0.1602
Total plaque >0 mm^3^, n (%)	19 (25.0%)	4 (33.3%)	5 (45.4%)	1 (100.0%)	0.1602
Total plaque <20 mm^3^, n (%)	68 (89.5%)	11 (91.7%)	9 (81.8%)	1 (100.0%)	0.7699
Total plaque ≥20 mm^3^, n (%)	8 (10.5%)	1 (8.3%)	2 (18.2%)	0 (0.0%)	0.7699
Total plaque	0.0 (0.0, 0.5)	0.0 (0.0, 3.5)	0.0 (0.0, 4.0)	11.0 (11.0, 11.0)	0.2835
Calcified plaque	0.0 (0.0, 0.0)	0.0 (0.0, 0.0)	0.0 (0.0, 0.0)	0.0 (0.0, 0.0)	0.8877
Low-attenuation plaque	0.0 (0.0, 0.0)	0.0 (0.0, 0.0)	0.0 (0.0, 0.0)	0.0 (0.0, 0.0)	0.9071
High risk low-attenuation plaque (≥10 mm^3^)	1 (1.5%)	0 (0.0%)	0 (0.0%)	0 (0.0%)	1.0000
Noncalcified plaque	0.0 (0.0, 0.5)	0.0 (0.0, 3.5)	0.0 (0.0, 4.0)	11.0 (11.0, 11.0)	0.2835
Left main					
Total plaque	0.0 (0.0, 0.0)	0.0 (0.0, 0.0)	0.0 (0.0, 0.0)	0.0 (0.0, 0.0)	0.9570
Calcified plaque	0.0 (0.0, 0.0)	0.0 (0.0, 0.0)	0.0 (0.0, 0.0)	0.0 (0.0, 0.0)	1.0000
Low-attenuation plaque	0.0 (0.0, 0.0)	0.0 (0.0, 0.0)	0.0 (0.0, 0.0)	0.0 (0.0, 0.0)	1.0000
Noncalcified plaque	0.0 (0.0, 0.0)	0.0 (0.0, 0.0)	0.0 (0.0, 0.0)	0.0 (0.0, 0.0)	0.9570
Left anterior descending artery					
Total plaque	0.0 (0.0, 0.0)	0.0 (0.0, 0.0)	0.0 (0.0, 0.0)	0.0 (0.0, 0.0)	0.9302
Calcified plaque	0.0 (0.0, 0.0)	0.0 (0.0, 0.0)	0.0 (0.0, 0.0)	0.0 (0.0, 0.0)	1.0000
Low-attenuation plaque	0.0 (0.0, 0.0)	0.0 (0.0, 0.0)	0.0 (0.0, 0.0)	0.0 (0.0, 0.0)	0.9391
Noncalcified plaque	0.0 (0.0, 0.0)	0.0 (0.0, 0.0)	0.0 (0.0, 0.0)	0.0 (0.0, 0.0)	0.9302
Left circumflex artery					
Total plaque	0.0 (0.0, 0.0)	0.0 (0.0, 0.0)	0.0 (0.0, 0.0)	0.0 (0.0, 0.0)	0.9333
Calcified plaque	0.0 (0.0, 0.0)	0.0 (0.0, 0.0)	0.0 (0.0, 0.0)	0.0 (0.0, 0.0)	1.0000
Low-attenuation plaque	0.0 (0.0, 0.0)	0.0 (0.0, 0.0)	0.0 (0.0, 0.0)	0.0 (0.0, 0.0)	0.9570
Noncalcified plaque	0.0 (0.0, 0.0)	0.0 (0.0, 0.0)	0.0 (0.0, 0.0)	0.0 (0.0, 0.0)	0.9333
Right coronary artery					
Total plaque	0.0 (0.0, 0.0)	0.0 (0.0, 0.0)	0.0 (0.0, 4.0)	11.0 (11.0, 11.0)	**0.0138**
Calcified plaque	0.0 (0.0, 0.0)	0.0 (0.0, 0.0)	0.0 (0.0, 0.0)	0.0 (0.0, 0.0)	0.8877
Low-attenuation plaque	0.0 (0.0, 0.0)	0.0 (0.0, 0.0)	0.0 (0.0, 0.0)	0.0 (0.0, 0.0)	0.4120
Noncalcified plaque	0.0 (0.0, 0.0)	0.0 (0.0, 0.0)	0.0 (0.0, 4.0)	11.0 (11.0, 11.0)	**0.0128**

Median (IQR), plaque volume is reported in mm^3^.

**Bold** value indicates statistically significant *P* values (*P* < 0.05).

Abbreviations as in Tables 1 and 2.

#### 30-year PREVENT cohort

Among individuals with CAC = 0, 30-year PREVENT risk stratification plaque burden was similar (Supplemental Table 4). Plaque volumes were very low across borderline-, intermediate-, and high-risk groups.

### Distribution of plaque severity across risk strata

We classified each CTA into HeartFlow plaque stages (0, mild [1-100 mm^3^], moderate [101-250 mm^3^], severe [251-750 mm^3^], and extensive [>750 mm^3^]) and cross-tabulated these with risk categories (Table 7). In the ASCVD cohort, 63 of 128 low-risk participants (49.2%) had no detectable plaque and 39 (30.5%) had only mild plaque; however, 26 (20%) had moderate-to-extensive plaque (volume ≥101 mm^3^). In the PREVENT 10-year cohort, 63 of 116 low-risk patients (54%) had no plaque, 36 (31%) had mild plaque, and 17 (15%) had moderate or greater plaque. In the borderline-risk groups, moderate-to-extensive plaque was present in 18 of 40 ASCVD patients (45%) and 11 of 32 PREVENT patients (34%). The prevalence of moderate or greater plaque rose to 52.3% and 56.5% among intermediate-risk ASCVD and PREVENT groups, respectively, and to 73.1% and 81.2% in high-risk groups. With 30-year PREVENT, moderate-to-extensive plaque was virtually absent among low- and borderline-risk individuals, occurred in 13% of intermediate-risk stratum, and was seen in 46.4% of high-risk individuals (all *P* < 0.0001). These distributions underscore that significant plaque burden is not confined to high-risk categories and that plaque quantification can identify SA patients with substantial disease despite low predicted risk.

**Table 7:** Distribution of HeartFlow Plaque Staging Across 10-Year ASCVD and 10-Y and 30-Y PREVENT Risk Categories

	HeartFlow Plaque Staging					*P* Value
	0	Mild (TPV 1–100 mm^3^)	Moderate (TPV 101–250 mm^3^)	Severe (TPV 251–750 mm^3^)	Extensive (TPV >750 mm^3^)	
10-y ASCVD cohort, N	81	85	40	43	25	
10-y ASCVD risk, n (%)						**<0.0001**
Low risk (<5%)	63 (77.8%)	39 (45.9%)	12 (30.0%)	11 (25.6%)	3 (12.0%)	
Borderline risk (5%–7.4%)	9 (11.1%)	13 (15.3%)	7 (17.5%)	4 (9.3%)	7 (28.0%)	
Intermediate risk (7.5%–19.9%)	8 (9.9%)	23 (27.1%)	8 (20.0%)	21 (48.8%)	5 (20.0%)	
High risk (≥20%)	1 (1.2%)	10 (11.8%)	13 (32.5%)	7 (16.3%)	10 (40.0%)	
10-y PREVENT cohort, N	80	73	30	31	19	
10-y PREVENT risk, n (%)						**<0.0001**
Low risk (<5%)	63 (78.8%)	36 (49.3%)	9 (30.0%)	6 (19.4%)	2 (10.5%)	
Borderline risk (5%–7.4%)	9 (11.3%)	12 (16.4%)	4 (13.3%)	3 (9.7%)	4 (21.1%)	
Intermediate risk (7.5%–19.9%)	8 (10.0%)	22 (30.1%)	10 (33.3%)	20 (64.5%)	9 (47.4%)	
High risk (≥20%)	0 (0.0%)	3 (4.1%)	7 (23.3%)	2 (6.5%)	4 (21.1%)	
30-y PREVENT cohort, N	80	73	30	31	19	
30-y PREVENT risk, n (%)						**<0.0001**
Low risk (<5%)	0 (0.0%)	2 (2.7%)	0 (0.0%)	0 (0.0%)	0 (0.0%)	
Borderline risk (5%–7.4%)	5 (6.3%)	1 (1.4%)	0 (0.0%)	0 (0.0%)	0 (0.0%)	
Intermediate risk (7.5%–19.9%)	44 (55.0%)	19 (26.0%)	5 (16.7%)	2 (6.5%)	2 (10.5%)	
High risk (≥20%)	31 (38.8%)	51 (69.9%)	25 (83.3%)	29 (93.5%)	17 (89.5%)	

**Bold** value indicates statistically significant *P* values (*P* < 0.05).

Abbreviations as in Tables 1 and 2.

Reverse cross-tabulation of Table 7 showed that among individuals with moderate plaque (101-250 mm^3^), 30.0% (n = 12/40) of ASCVD-defined and 30.0% (n = 9/30) of PREVENT-defined cohorts were classified as low risk, whereas 17.5% (n = 7/40) and 13.3% (n = 4/30), respectively, were classified as borderline risk. Overall, 47.5% (n = 19/40) of ASCVD-defined and 43.3% (n = 13/30) of PREVENT-defined individuals with moderate plaque remained below the intermediate-risk category. Among those with moderate-to-extensive plaque, 40.7% (n = 44/108) and 35.0% (n = 28/80), respectively, were classified as low or borderline risk.

## Discussion

In this retrospective analysis of the DILWALE registry, we report several key findings (Central Illustration). First, a substantial proportion of participants were intermediate or high-risk by PREVENT and ASCVD scores. Second, higher 10-year PREVENT and ASCVD risk categories were associated with progressively greater coronary plaque prevalence and volumes across all plaque types. The 30-year PREVENT model demonstrated comparable trends, with the highest burden of CP, NCP, and LAP in high-risk categories. Third, almost 1 in 10 SA adults had detectable plaque despite the absence of calcification. The 30-year PREVENT model revealed greater plaque prevalence across higher-risk categories even among individuals with CAC = 0. Collectively, these findings show that PREVENT and ASCVD scores demonstrate a graded association with quantitative plaque burden in this cohort. They further indicate that CAC may fail to detect noncalcified atherosclerosis identified by CCTA. However, our study evaluates the relationship and discordance between predicted cardiovascular risk and plaque burden measured on CCTA, rather than clinical cardiovascular outcomes. Coronary plaque burden represents an imaging marker associated with future risk, not a clinical outcome itself, and the absence of longitudinal outcomes precludes assessment of whether plaque-risk discordance translates into differences in event rates.

**Central Illustration:**
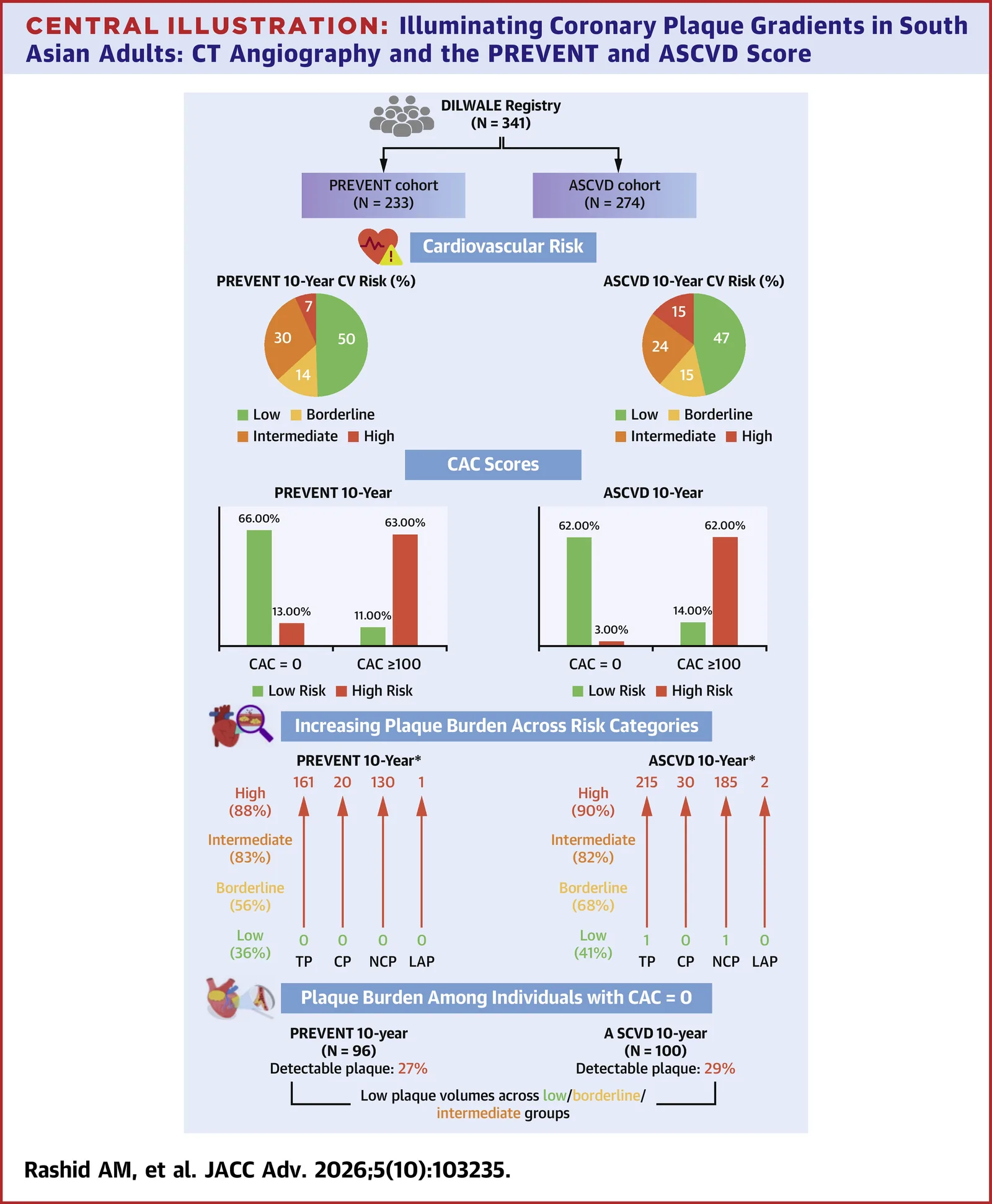
Illuminating Coronary Plaque Gradients in South Asian Adults: CT Angiography and the PREVENT and ASCVD Score Central illustration summarizing the main findings that coronary computed tomography angiography–measured plaque burden rises across higher Pooled Cohort Equations and PREVENT-risk categories, with higher NCP volumes observed in higher-risk groups. ASCVD = atherosclerotic cardiovascular disease; CAC = coronary artery calcium; CP = calcified plaque; CT = computed tomography; CV = cardiovascular; DILWALE = DIL Wellness and Arterial Health Longitudinal Evaluation; LAP = low-attenuation plaque; NCP = noncalcified plaque; TP = total plaque.

The HeartFlow plaque staging showed substantial plaque in some patients labeled low or borderline risk. Approximately 1 in 5 ASCVD low-risk participants and 1 in 6 PREVENT low-risk participants had moderate-to-extensive plaque volumes. Similarly, about one-third of borderline-risk individuals had moderate or worse plaque. Reverse analysis showed that substantial anatomic plaque persisted in low- and borderline-risk categories. These observations suggest that plaque imaging may capture differences in coronary atherosclerosis that are not fully reflected by traditional risk scores alone because volumetric CTA measures plaque burden rather than predicted future risk. However, the absence of outcomes data and a comparator population prevents us from determining whether the calculators underestimated future event risk.

Our results show that a substantial proportion of SA adults have significant coronary plaque burden despite low or borderline predicted risk, extending prior observations. The UK Biobank study of over 450,000 participants (including >8,000 SA) found that ASCVD events were 2-fold higher in SA adults than in European adults over a median 11-year follow-up.^2^ The QRISK3 scores were 1.4-fold and PCE scores were 1.1-fold higher in SA than in White people in the United Kingdom, yet they still underestimated the true risk in SA. Similarly, another study showed that PCE underestimated ASCVD risk (mean predicted risk vs observed risk: 1.8% vs 4.9%) among 1835 SA adults.^16^ Because the PCE does not include a specific category for SA individuals, participants in our study were classified using the available White/other category, consistent with routine clinical use of the calculator.^17^ This limitation was one reason for comparing PCE with the newer PREVENT equations, which do not use race as a model variable. The newer PREVENT equations, derived from >6.5 million U.S. adults, improves risk assessment by incorporating health measures such as estimated glomerular filtration rate, urine albumin-to-creatinine ratio (UACR), and glycated hemoglobin.^17^ In our cohort, PREVENT equations align with plaque burden in SA, supporting its relevance in this higher-risk group. Yet, PREVENT forecast events, not actual disease burden.^18^

CCTA-measured plaque reflects existing anatomic disease burden, whereas risk scores estimate future event risk. In our cohort, NCP had the highest plaque burden by risk category, and LAP prevalence rose with higher 10-year PREVENT or PCE-risk scores. Analysis of SCOT-HEART Trial (Scottish Computed Tomography of the HEART), enrolling 1769 patients over 4.7 years, showed that LAP was strongly associated with stenosis severity and CAC score (r = 0.83 and r = 0.62, respectively).^19^ LAP burden was the strongest predictor of MI (HR: 1.60 per doubling) irrespective of stenosis severity, CAC, or Framingham-derived risk score.^19^ Notably, LAP burden >4% conferred ∼5-fold higher MI-risk. Similarly, in 1,469 low-to-intermediate-risk patients, LAP <60 HU was the strongest major adverse cardiac events predictor (HR: 4.96; 95% CI: 2.0-12.2), whereas stenosis severity and plaque type (HR: 1.5; 95% CI: 1.1-2.3 and HR 1.7: 95% CI: 1.1-2.6) were less powerful.^20^ In a matched CCTA cohort of 165 SA individuals, TP percent atheroma volume (PAV) (11.9% vs 6.2%), NCP PAV (5.1% vs 3.8%), and CP PAV (4.9% vs 2.4%) were all higher than in matched non-Hispanic White individuals.^18^ Results were consistent in individuals <55 years. Recognizing computed tomography angiography–based reproducible, automated volumetric analysis of plaque and its strong concordance with intravascular ultrasound, imaging guidelines from Society of Cardiovascular Computed Tomography and North American Society of Cardiovascular Imaging support its use for standardized risk assessment.^21^

The CAC = 0 findings are descriptive, as the small size of high-risk subgroups limits interpretability. Among patients with CAC = 0, approximately 10% had plaque meeting prespecified threshold; however, this proportion is uncertain in the highest-risk strata due to very small sample sizes. In the MASALA registry, 30% of those at intermediate 10-year PCE risk had CAC = 0, whereas 37% had CAC >100.^22^ Another SA cohort found NCP on CCTA in 28.8% of patients with CAC = 0, higher than the ∼10% typically seen in Western populations.^23^^,^^24^ Hence, atherosclerosis in SA individuals can advance significantly before calcification appears. Consequently, the 5-year “warranty period” of CAC = 0 in Western populations may not be reliably applied to SA.^25^ For SA, the absence of calcium is not the absence of disease. CCTA directly measures plaque burden and can identify high-risk patients whom traditional risk scores and CAC would otherwise misclassify as low risk.

Higher estimated risk aligns with greater HeartFlow plaque stage, consistent with previous CCTA studies showing that low-attenuation, lipid-rich plaque predicts future MI.^19^ SA cohorts show earlier onset and higher NCP and TP than other groups.^26^ This may explain why higher HeartFlow stages concentrate among patients labeled intermediate-to-high-risk in our sample. In contrast, a meta-analysis of asymptomatic cohorts shows a lower NCP prevalence with CAC = 0 (∼10%), but symptomatic and some ethnicity-specific cohorts report higher rates (∼28%).^11^^,^^23^ Recent AI-enabled volumetric plaque tools correlate well with intravascular ultrasound and allow reproducible quantification of TP, CP, and NCP across sites, validating stage-based HeartFlow assessment.^14^ However, these tools still require broader cross-platform validation of outcomes before routine prognostic use.

These findings may influence risk stratification in SA individuals. Current ACC/AHA guidelines list SA ancestry as risk-enhancing and recommend CAC to refine intermediate risk.^27^ A CAC score of zero can defer statins for many.^28^ However, our data suggest that moderate plaque burden in low- or borderline-risk patients may warrant consideration of intensified lifestyle modification and lipid-lowering therapy, rather than reassurance based solely on the calculated score. Clinicians should individualize care by integrating low-density lipoprotein-cholesterol (LDL-C) levels, diabetes status, blood pressure, symptoms, medications, and patient preferences. This aligns with the 2026 ACC/AHA Multisociety Dyslipidemia Guidelines, which recommend earlier lipid-lowering therapy, selective CAC measurement, and lower LDL-C targets by risk.^29^ Our results neither establish a treatment threshold nor confirm that anatomy-guided escalation improves outcomes. These findings align with expert recommendations such as Coronary Artery Disease Reporting–Data System (CAD-RADS 2.0), which support routine plaque reporting and AI-based quantitative analysis to standardize assessments.^21^^,^^30^^,^^31^

These findings carry direct implications for preventive decision-making in SA adults. Current ACC/AHA guidelines recommend lifestyle modification for low-risk (<5%) individuals and statin consideration for borderline-risk (5–<7.5%) only after shared decision-making based on risk-enhancing factors, including SA ancestry. The HeartFlow DECIDE plaque-staging framework links moderate-to-extensive plaque (≥101 mm^3^) to high-intensity statin therapy, offering an anatomy-based treatment approach. Here, 20% of ASCVD-defined and 15% of PREVENT-defined low-risk participants in our cohort would qualify for intensified therapy, despite low-risk scores. Among borderline-risk, 45% (ASCVD) and 34% (PREVENT) met this plaque threshold. This highlights a gap between risk scores and actual anatomic disease. Statin use remains individualized based on LDL-C, diabetes, concurrent medications, and patient-preferences, but the presence of subclinical noncalcified atherosclerosis in low/borderline-risk individuals, especially where CAC underestimates disease, suggests anatomy-informed intervention may help prevent events. Prospective studies in SA cohorts linking plaque metrics to outcomes are needed to set evidence-based thresholds for prevention and to determine if adding CCTA-derived plaque staging to SA-specific prevention improves outcomes (Figure 3).

**Figure 3:**
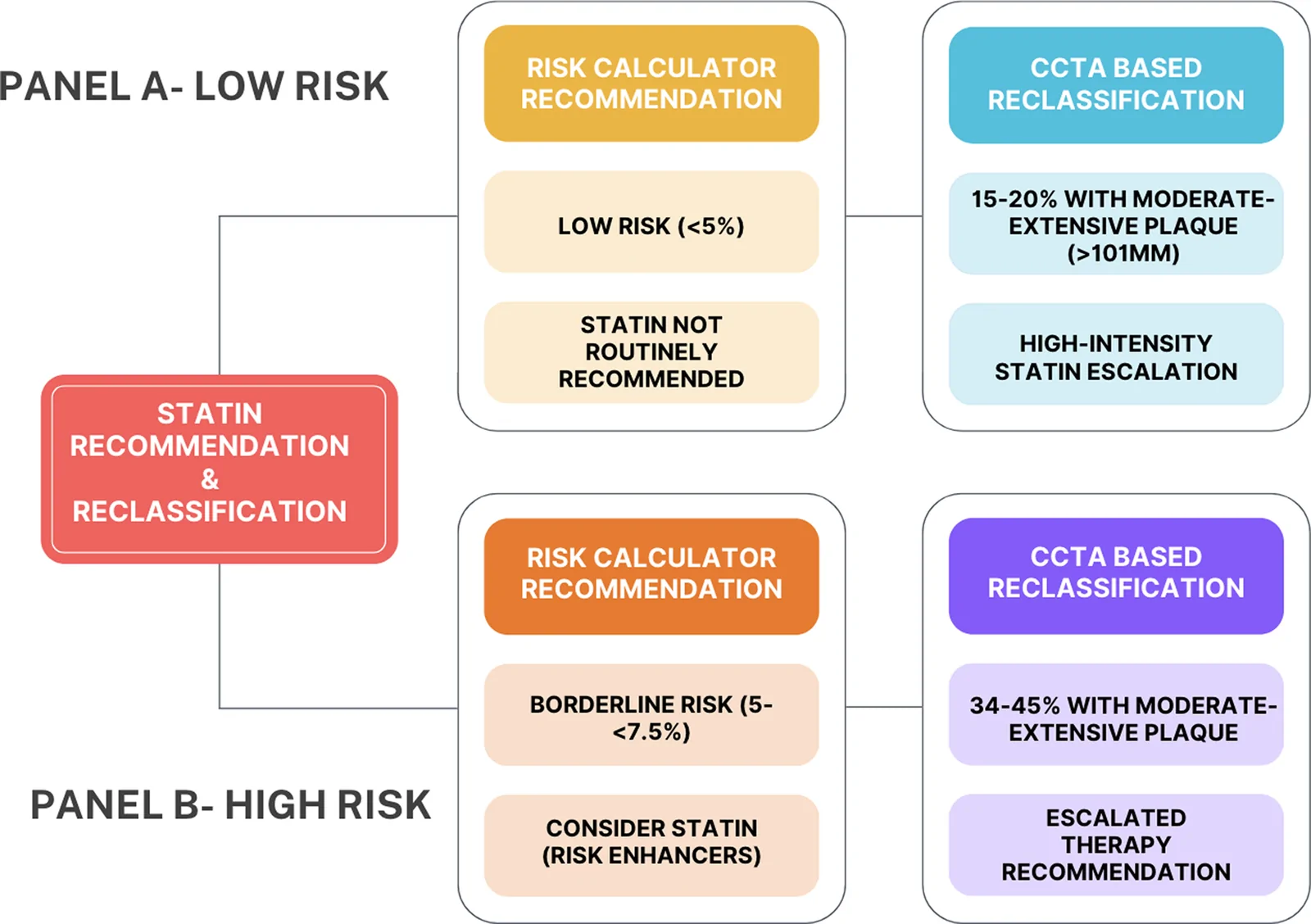
Proposed Prevention Framework Integrating CCTA Plaque Staging With Traditional Risk Scores in South Asian Adults Proportion of low (A) and borderline risk (B) individuals with plaque ≥101 mm^3^ in the DILWALE cohort. CCTA = coronary computed tomography angiography.

In summary, among the clinically referred SA adults, higher PREVENT and ASCVD/PCE-risk categories were linked to greater plaque burden, even in those with CAC = 0. Reverse cross-tabulation also showed that many patients with moderate or greater plaque still fell into low- or borderline-risk categories. Quantitative plaque analysis directly measures anatomic disease burden and reveals subclinical noncalcified disease often missed by CAC. Therefore, the absence of calcium does not equate to the absence of disease in SA. Incorporating quantitative CCTA into risk assessment may refine anatomic stratification and help inform preventive strategies in selected SA patients.

### Study Limitations

First, the retrospective design and absence of longitudinal follow-up precluded the assessment of whether plaque burden or risk-score discordance translated into future ASCVD or different clinical events.^19^ Second, we did not perform a head-to-head comparison between PCE and PREVENT because our objective was to evaluate each risk score’s association with coronary plaque burden, not compare their relative performance. Moreover, plaque burden was an imaging endpoint rather than a clinical outcome. Third, CAC = 0 subgroups, particularly high-risk individuals, were very small (n = 2 for PREVENT and n = 1 for PCE), with no significant differences in plaque volumes observed across strata (*P* > 0.10 for all). Although the presence of measurable NCP in CAC = 0 is clinically relevant, its prognostic significance requires confirmation in larger cohorts. Thus, these findings are hypothesis-generating. Fourth, variation in LAP thresholds across studies complicates direct comparison.^19^^,^^32^ In addition, we applied a conservative TPV threshold of <20 mm^3^ to reduce false-positive plaque detection from automated analysis. This prespecified cutoff was applied uniformly across the cohort. We additionally reported plaque prevalence using TPV = 0 mm^3^, TPV >0 mm^3^, TPV <20 mm^3^, and TPV ≥20 mm^3^ to show the impact of alternative plaque definitions. However, several CAC = 0 risk strata were small, which may have limited estimate precision. Furthermore, statin use increased across risk strata, potentially attenuating the observed plaque gradient. Nonrandom statin exposure and limited sample size precluded adjustment for multiple risk groups despite statin-stratified analysis; thus, residual confounding may persist. Moreover, we analyzed the risk categories to evaluate the overall graded association with plaque burden rather than multiple pairwise comparisons, because the middle-risk categories were small. Risk score component availability was also incomplete, particularly for the 10-year PREVENT risk group, and missingness may not have been random. Although we performed sensitivity analyses comparing plaque burden by score availability, we did not perform multiple imputation; hence, residual bias cannot be excluded. In addition, because our study did not include a non-SA comparison group, we cannot determine whether the observed plaque-risk discordance differs from other populations. Finally, this single-state, clinically referred CCTA cohort likely introduces referral and selection bias, inflating plaque prevalence and limiting generalizability to asymptomatic or community-based SA populations.

Perspectives **COMPETENCY IN MEDICAL KNOWLEDGE:** In this clinically referred SA cohort, PREVENT and PCE risk scores were associated with plaque burden. CCTA identified moderate-to-extensive plaque in 20% of ASCVD-defined and 15% of PREVENT-defined low-risk individuals. In addition, 47.5% of ASCVD-defined and 43.3% of PREVENT-defined individuals with moderate plaque remained below intermediate risk. These findings suggest that quantitative CCTA may refine anatomic risk in selected SA patients by identifying subclinical NCP that CAC can miss.

**TRANSLATIONAL OUTLOOK:** Future studies should determine whether quantitative plaque staging adds prognostic value beyond PREVENT, PCE, and CAC in prospective, community-based SA cohorts. Research should also assess how referral bias, statin exposure, and the conservative TPV <20 mm^3^ threshold affect clinical translation. Studies should also evaluate whether plaque-guided prevention improves outcomes.

## Funding support and author disclosures

Dr Khan has received consulting fees from Bayer, Novartis, and Boehringer Ingelheim. All other authors have reported that they have no relationships relevant to the contents of this paper to disclose.
