# Supplementary material for: Coronary Plaque Burden in South Asian Adults: Insights From the DILWALE Registry
Source: JACC Adv. 2026 Sep 18;5(10):103235. doi: 10.1016/j.jacadv.2026.103235 (PMC13627118; doi:10.1016/j.jacadv.2026.103235)
Supplement: Supplementary file 1 [file mmc1.docx]

**Supplementary Table 1: Overlap and Concordance Between the 10-Year ASCVD/PCE and 10-Year AHA PREVENT Risk Categories**

| **10-year ASCVD/PCE Risk** | **10-year AHA PREVENT Risk** | | | | |  |
| --- | --- | --- | --- | --- | --- | --- |
|  | **Missing** | **Low-risk**  **(< 5%)** | **Borderline risk**  **(5% to 7.4%)** | **Intermediate risk**  **(7.5% to 19.9%)** | **High risk**  **(≥ 20%)** | **Total** |
| **Missing** | 43 | 17 | 0 | 1 | 6 | 67 |
| **Low-risk**  **(< 5%)** | 23 | 94 | 10 | 1 | 0 | 128 |
| **Borderline risk**  **(5% to 7.4%)** | 10 | 4 | 18 | 8 | 0 | 40 |
| **Intermediate risk**  **(7.5% to 19.9%)** | 18 | 1 | 4 | 41 | 1 | 65 |
| **High risk**  **(≥ 20%)** | 14 | 0 | 0 | 18 | 9 | 41 |
| **Total** | 108 | 116 | 32 | 69 | 16 | 341 |

**Values are reported as N**

AHA, American Heart Association; ASCVD, Atherosclerotic cardiovascular disease; PCE, Pooled cohort equations

**Supplementary Table 2: Presence of Plaque and Plaque Subtypes Among Patients Stratified by the 30-Year PREVENT Calculator**

|  | **30-Year PREVENT Risk** | | | |  |
| --- | --- | --- | --- | --- | --- |
|  | **Low risk (< 5%)** | **Borderline risk (5% to 7.4%)** | **Intermediate risk (7.5% to 19.9%)** | **High risk (>= 20%)** | **P-value** |
|  | **(N=2)** | **(N=6)** | **(N=72)** | **(N=153)** |  |
| **Overall Coronary Arteries** |  |  |  |  |  |
| **Presence of Plaque, n (%)** | 1 (50%) | 1 (17%) | 20 (28%) | 109 (71%) | **<0.0001** |
| **Total Plaque = 0 mm^3^, n (%)** | 0 (0.0%) | 5 (83.3%) | 44 (61.1%) | 31 (20.3%) | **<0.0001** |
| **Total Plaque >0 mm^3^, n (%)** | 2 (100.0%) | 1 (16.7%) | 28 (38.9%) | 122 (79.7%) | **<0.0001** |
| **Total Plaque <20 mm^3^, n (%)** | 1 (50.0%) | 5 (83.3%) | 52 (72.2%) | 44 (28.8%) | **<0.0001** |
| **Total Plaque ≥20 mm^3^, n (%)** | 1 (50.0%) | 1 (16.7%) | 20 (27.8%) | 109 (71.2%) | **<0.0001** |
| **Total Plaque** | 12.0  (1.0, 23.0) | 0.0  (0.0, 0.0) | 0.0  (0.0, 25.0) | 88.0  (8.0, 378.0) | **<0.0001** |
| **Calcified Plaque** | 0.0  (0.0, 0.0) | 0.0  (0.0, 0.0) | 0.0  (0.0, 2.0) | 10.0  (0.0, 71.0) | **<0.0001** |
| **Low attenuation Plaque** | 0.0  (0.0, 0.0) | 0.0  (0.0, 0.0) | 0.0  (0.0, 1.0) | 1.0  (0.0, 4.0) | **<0.0001** |
| **High risk Low attenuation plaque (≥10mm3)** | 0 (0.0%) | 0 (0.0%) | 2 (3.6%) | 19 (22.1%) | **0.0127** |
| **Non-Calcified Plaque** | 12.0  (1.0, 23.0) | 0.0  (0.0, 0.0) | 0.0  (0.0, 24.0) | 79.0  (8.0, 314.0) | **<0.0001** |
|  |  |  |  |  |  |
| **Left Main** |  |  |  |  |  |
| **Total plaque** | 0.0  (0.0, 0.0) | 0.0  (0.0, 0.0) | 0.0  (0.0, 0.0) | 0.0  (0.0, 11.0) | **0.0001** |
| **Calcified Plaque** | 0.0  (0.0, 0.0) | 0.0  (0.0, 0.0) | 0.0  (0.0, 0.0) | 0.0  (0.0, 1.0) | **0.0003** |
| **Low Attenuation Plaque** | 0.0  (0.0, 0.0) | 0.0  (0.0, 0.0) | 0.0  (0.0, 0.0) | 0.0  (0.0, 0.0) | 0.1968 |
| **Non-Calcified Plaque** | 0.0  (0.0, 0.0) | 0.0  (0.0, 0.0) | 0.0  (0.0, 0.0) | 0.0  (0.0, 9.0) | **0.0001** |
|  |  |  |  |  |  |
| **Left Anterior Descending Artery** |  |  |  |  |  |
| **Total Plaque** | 0.0  (0.0, 0.0) | 0.0  (0.0, 0.0) | 0.0  (0.0, 8.0) | 34.0  (0.0, 144.0) | **<0.0001** |
| **Calcified Plaque** | 0.0  (0.0, 0.0) | 0.0  (0.0, 0.0) | 0.0  (0.0, 0.0) | 3.0  (0.0, 22.0) | **<0.0001** |
| **Low Attenuation Plaque** | 0.0  (0.0, 0.0) | 0.0  (0.0, 0.0) | 0.0  (0.0, 0.0) | 0.0  (0.0, 2.0) | **<0.0001** |
| **Non-Calcified Plaque** | 0.0  (0.0, 0.0) | 0.0  (0.0, 0.0) | 0.0  (0.0, 8.0) | 30.0  (0.0, 120.0) | **<0.0001** |
|  |  |  |  |  |  |
| **Left Circumflex Artery** |  |  |  |  |  |
| **Total plaque** | 12.0 (1.0, 23.0) | 0.0 (0.0, 0.0) | 0.0 (0.0, 0.0) | 2.0 (0.0, 59.0) | **<0.0001** |
| **Calcified Plaque** | 0.0 (0.0, 0.0) | 0.0 (0.0, 0.0) | 0.0 (0.0, 0.0) | 0.0 (0.0, 7.0) | **<0.0001** |
| **Low Attenuation Plaque** | 0.0 (0.0, 0.0) | 0.0 (0.0, 0.0) | 0.0 (0.0, 0.0) | 0.0 (0.0, 1.0) | **0.1078** |
| **Non-Calcified Plaque** | 12.0 (1.0, 23.0) | 0.0 (0.0, 0.0) | 0.0 (0.0, 0.0) | 2.0 (0.0, 52.0) | **<0.0001** |
|  |  |  |  |  |  |
| **Right Coronary Artery** |  |  |  |  |  |
| **Total Plaque** | 0.0  (0.0, 0.0) | 0.0  (0.0, 0.0) | 0.0  (0.0, 0.0) | 7.0  (0.0, 72.0) | **<0.0001** |
| **Calcified Plaque** | 0.0  (0.0, 0.0) | 0.0  (0.0, 0.0) | 0.0  (0.0, 0.0) | 0.0  (0.0, 9.0) | **0.0005** |
| **Low Attenuation Plaque** | 0.0  (0.0, 0.0) | 0.0  (0.0, 0.0) | 0.0  (0.0, 0.0) | 0.0  (0.0, 1.0) | **0.0329** |
| **Non-Calcified Plaque** | 0.0  (0.0, 0.0) | 0.0  (0.0, 0.0) | 0.0  (0.0, 0.0) | 7.0  (0.0, 65.0) | **<0.0001** |

**Median plaque volume is reported in mm^3^**

IQR, Interquartile range

**Supplementary Table 3: Comparison of Coronary Plaque Prevalence According to Availability of 10-Year ASCVD/PCE and 10-Year AHA PREVENT Risk Scores**

| **Complete HeartFlow Cohort**  **(N = 341)** | | | | | | |
| --- | --- | --- | --- | --- | --- | --- |
| **Plaque Definition** | **ASCVD Risk Data Unavailable**  **(N = 67)** | **ASCVD Risk Data Available**  **(N = 274)** | **P-value** | **PREVENT Risk Data Unavailable**  **(N = 108)** | **PREVENT Risk Data Available**  **(N = 233)** | **P-value** |
| **Any detectable plaque (TPV >0 mm³), n (%)** |  |  |  |  |  |  |
| No | 15 (22.4) | 81 (29.6) | 0.2418 | 16 (14.8) | 80 (34.3) | **0.0002** |
| Yes | 52 (77.6) | 193 (70.4) |  | 92 (85.2) | 153 (65.7) |  |
| **Total plaque (TPV ≥20 mm³), n (%)** |  |  |  |  |  |  |
| No | 21 (31.3) | 105 (38.3) | 0.2888 | 24 (22.2) | 102 (43.8) | **0.0001** |
| Yes | 46 (68.7) | 169 (61.7) |  | 84 (77.8) | 131 (56.2) |  |
|  |  |  |  |  |  |  |
| **Sensitivity Analysis Excluding Patients Missing Both Risk Scores**  **(N = 298)** | | | | | | |
| **Plaque Definition** | **ASCVD Risk Data Unavailable**  **(N = 24)** | **ASCVD Risk Data Available**  **(N = 274)** | **P-value** | **PREVENT Risk Data Unavailable**  **(N = 65)** | **PREVENT Risk Data Available**  **(N = 233)** | **P-value** |
| **Any detectable plaque (TPV >0 mm³), n (%)** |  |  |  |  |  |  |
| No | 10 (41.7) | 81 (29.6) | 0.217 | 11 (16.9) | 80 (34.3) | **0.0070** |
| Yes | 14 (58.3) | 193 (70.4) |  | 54 (83.1) | 153 (65.7) |  |
| **Total plaque (TPV ≥20 mm³), n (%)** |  |  |  |  |  |  |
| No | 13 (54.2) | 105 (38.3) | 0.128 | 16 (24.6) | 102 (43.8) | **0.0052** |
| Yes | 11 (45.8) | 169 (61.7) |  | 49 (75.4) | 131 (56.2) |  |

AHA, American Heart Association; ASCVD, Atherosclerotic cardiovascular disease; PCE, Pooled cohort equations; TPV, Total plaque volume.

**Supplementary Table 4: Presence of Plaque and Plaque Subtypes Among Patients with a Calcium score of 0 Agatston Units Stratified by the 30-Year AHA PREVENT Calculator**

|  | **30-Year PREVENT Risk** | | | |  |
| --- | --- | --- | --- | --- | --- |
|  | **Low risk (< 5%)** | **Borderline risk (5% to 7.4%)** | **Intermediate risk (7.5% to 19.9%)** | **High risk (>= 20%)** | **P-value** |
|  | **(N=2)** | **(N=5)** | **(N=48)** | **(N=41)** |  |
|  |  |  |  |  |  |
| **Overall Coronary Arteries** |  |  |  |  |  |
| **Presence of Plaque**, n (%) | 1 (50%) | 0 (0%) | 2 (4%) | 6 (15%) | 0.0715 |
| **Total Plaque = 0 mm^3^, n (%)** | 0 (0.0%) | 5 (100.0%) | 41 (85.4%) | 24 (58.5%) | **0.0015** |
| **Total Plaque >0 mm^3^, n (%)** | 2 (100.0%) | 0 (0.0%) | 7 (14.6%) | 17 (41.5%) | **0.0015** |
| **Total Plaque <20 mm^3^, n (%)** | 1 (50.0%) | 5 (100.0%) | 46 (95.8%) | 35 (85.4%) | 0.0715 |
| **Total Plaque ≥20 mm^3^, n (%)** | 1 (50.0%) | 0 (0.0%) | 2 (4.2%) | 6 (14.6%) | 0.0715 |
| **Total Plaque** | 12.0  (1.0, 23.0) | 0.0  (0.0, 0.0) | 0.0  (0.0, 0.0) | 0.0  (0.0, 4.0) | **0.0038** |
| **Calcified Plaque** | 0.0  (0.0, 0.0) | 0.0  (0.0, 0.0) | 0.0  (0.0, 0.0) | 0.0  (0.0, 0.0) | 0.568 |
| **Low Attenuation Plaque** | 0.0  (0.0, 0.0) | 0.0  (0.0, 0.0) | 0.0  (0.0, 0.0) | 0.0  (0.0, 0.0) | 0.2872 |
| **High risk Low Attenuation Plaque (≥10mm^3^)** | 0 (0.0%) | 0 (0.0%) | 0 (0.0%) | 1 (2.9%) | 0.4847 |
| **Non-Calcified Plaque** | 12.0  (1.0, 23.0) | 0.0  (0.0, 0.0) | 0.0  (0.0, 0.0) | 0.0  (0.0, 4.0) | **0.0038** |
| **Left Main** |  |  |  |  |  |
| **Total Plaque** | 0.0  (0.0, 0.0) | 0.0  (0.0, 0.0) | 0.0  (0.0, 0.0) | 0.0  (0.0, 0.0) | 0.8013 |
| **Calcified Plaque** | 0.0  (0.0, 0.0) | 0.0  (0.0, 0.0) | 0.0  (0.0, 0.0) | 0.0  (0.0, 0.0) | 1.0000 |
| **Low Attenuation Plaque** | 0.0  (0.0, 0.0) | 0.0  (0.0, 0.0) | 0.0  (0.0, 0.0) | 0.0  (0.0, 0.0) | 1.0000 |
| **Non-Calcified Plaque** | 0.0  (0.0, 0.0) | 0.0  (0.0, 0.0) | 0.0  (0.0, 0.0) | 0.0  (0.0, 0.0) | 0.8013 |
| **Left Anterior Descending Artery** |  |  |  |  |  |
| **Total Plaque** | 0.0  (0.0, 0.0) | 0.0  (0.0, 0.0) | 0.0  (0.0, 0.0) | 0.0  (0.0, 0.0) | 0.3041 |
| **Calcified Plaque** | 0.0  (0.0, 0.0) | 0.0  (0.0, 0.0) | 0.0  (0.0, 0.0) | 0.0  (0.0, 0.0) | 1.0000 |
| **Low Attenuation Plaque** | 0.0  (0.0, 0.0) | 0.0  (0.0, 0.0) | 0.0  (0.0, 0.0) | 0.0  (0.0, 0.0) | 0.0720 |
| **Non-Calcified Plaque** | 0.0  (0.0, 0.0) | 0.0  (0.0, 0.0) | 0.0  (0.0, 0.0) | 0.0  (0.0, 0.0) | 0.3041 |
| **Left Circumflex Artery** |  |  |  |  |  |
| **Total Plaque** | 12.0  (1.0, 23.0) | 0.0  (0.0, 0.0) | 0.0  (0.0, 0.0) | 0.0  (0.0, 0.0) | **0.0002** |
| **Calcified Plaque** | 0.0  (0.0, 0.0) | 0.0  (0.0, 0.0) | 0.0  (0.0, 0.0) | 0.0  (0.0, 0.0) | 1.0000 |
| **Low Attenuation Plaque** | 0.0  (0.0, 0.0) | 0.0  (0.0, 0.0) | 0.0  (0.0, 0.0) | 0.0  (0.0, 0.0) | 0.7193 |
| **Non-Calcified Plaque** | 12.0  (1.0, 23.0) | 0.0  (0.0, 0.0) | 0.0  (0.0, 0.0) | 0.0  (0.0, 0.0) | **0.0002** |
| **Right Coronary Artery** |  |  |  |  |  |
| **Total Plaque** | 0.0  (0.0, 0.0) | 0.0  (0.0, 0.0) | 0.0  (0.0, 0.0) | 0.0  (0.0, 0.0) | 0.3078 |
| **Calcified Plaque** | 0.0  (0.0, 0.0) | 0.0  (0.0, 0.0) | 0.0  (0.0, 0.0) | 0.0  (0.0, 0.0) | 0.5680 |
| **Low Attenuation Plaque** | 0.0  (0.0, 0.0) | 0.0  (0.0, 0.0) | 0.0  (0.0, 0.0) | 0.0  (0.0, 0.0) | 0.9108 |
| **Non-Calcified Plaque** | 0.0  (0.0, 0.0) | 0.0  (0.0, 0.0) | 0.0  (0.0, 0.0) | 0.0 (0.0, 3.0) | 0.3049 |

**Median plaque volume is reported in mm^3^**

IQR, Interquartile range
